# A Study of Platelet Inhibition, Using a ‘Point of Care’ Platelet Function Test, following Primary Percutaneous Coronary Intervention for ST-Elevation Myocardial Infarction [PINPOINT-PPCI]

**DOI:** 10.1371/journal.pone.0144984

**Published:** 2015-12-16

**Authors:** Thomas W. Johnson, Andrew D. Mumford, Lauren J. Scott, Stuart Mundell, Mark Butler, Julian W. Strange, Chris A. Rogers, Barnaby C. Reeves, Andreas Baumbach

**Affiliations:** 1 Bristol Heart Institute, Bristol, United Kingdom; 2 University of Bristol, Bristol, United Kingdom; Medical Faculty, Ludwig Maximilians University Munich, GERMANY

## Abstract

**Background:**

Rapid coronary recanalization following ST-elevation myocardial infarction (STEMI) requires effective anti-platelet and anti-thrombotic therapies. This study tested the impact of door to end of procedure (‘door-to-end’) time and baseline platelet activity on platelet inhibition within 24hours post-STEMI.

**Methods and Findings:**

108 patients, treated with prasugrel and procedural bivalirudin, underwent Multiplate® platelet function testing at baseline, 0, 1, 2 and 24hours post-procedure. Major adverse cardiac events (MACE), bleeding and stent thrombosis (ST) were recorded. Baseline ADP activity was high **(**88.3U [71.8–109.0]), procedural time and consequently bivalirudin infusion duration were short (median door-to-end time 55minutes [40–70] and infusion duration 30minutes [20–42]). Baseline ADP was observed to influence all subsequent measurements of ADP activity, whereas door-to-end time only influenced ADP immediately post-procedure. High residual platelet reactivity (HRPR ADP>46.8U) was observed in 75% of patients immediately post-procedure and persisted in 24% of patients at 2hours. Five patients suffered in-hospital MACE (4.6%). Acute ST occurred in 4 patients, all were <120mins post-procedure and had HRPR. No significant bleeding was observed. In a post-hoc analysis, pre-procedural morphine use was associated with significantly higher ADP activity following intervention.

**Conclusions:**

Baseline platelet function, time to STEMI treatment and opiate use all significantly influence immediate post-procedural platelet activity.

## Introduction

Optimal treatment of acute ST-elevation myocardial infarction (STEMI) requires early re-canalisation of the occluded coronary artery by primary percutaneous coronary intervention (PPCI) and passivation of coronary artery thrombosis with combined antiplatelet and anticoagulant drugs[1].

Prasugrel provides fast and effective platelet inhibition[2,3], although high baseline platelet activity, reduced drug bioavailability and concomitant use of opiates in the setting of STEMI may delay the antiplatelet effect of a loading dose[4,5]. Bivalirudin causes rapid thrombin inhibition and has a low bleeding risk. However, its short half-life is associated with an increased risk of acute stent thrombosis (ST) when treatment is limited to the peri-procedural period[6].

It was hoped that combined use of bivalirudin with prasugrel, a more potent oral P2Y12 antagonist than clopidogrel, would provide faster and more consistent inhibition of platelet activity, with improved clinical outcomes, particularly through minimizing the risk of acute ST[7]. Unfortunately BRAVE-4, a trial designed to test this combination of agents, was stopped prematurely due to slow recruitment and analysis of the outcomes for the 548 patients enrolled failed to show superiority of bivalirudin/prasugrel vs heparin/clopidogrel[8].

Acute STEMI pharmacotherapy is challenged by the potential conflict between shortening the time to revascularization and consequent reduction of exposure of patients to anti-platelet and anti-thrombotic therapy. PINPOINT-PPCI was designed to address these uncertainties. We aimed to profile platelet activity during the first 24 hours of treatment for STEMI using bivalirudin and prasugrel. Recent clinical data has highlighted an increased risk of acute ST in revascularized target lesions when bivalirudin is used, compared to unfractionated heparin monotherapy combined with potent oral P2Y12 inhibition[9]. Our study tests the interaction between procedural timing and acute antiplatelet and antithrombotic therapy, providing insights into the mechanisms driving acute ST with use of prasugrel and procedural bivalirudin.

## Methods

### Study design

The PINPOINT-PPCI study is a single-centre study of patients receiving anti-thrombotic treatment and PPCI for acute STEMI. The study was approved by a UK NHS Research Ethics Committee Number (REC: 10/H0106/87) on 20^th^ December 2010. The study was registered with Current Controlled Trials (www.controlled-trials.com/ISRCTN82257414), trial registration was completed following the commencement of patient recruitment due only to administrative delays. Patients presenting with acute STEMI between June 2011 & February 2013 were checked for eligibility and were invited to give verbal assent for participation at the time of emergency admission; patients were re-approached to give written consent after PPCI, within 24 hours of admission. The study protocol has been reported previously[10].

### Study population

Patients were eligible for the study if they presented to our regional heart attack unit with acute STEMI requiring PPCI. Patients were excluded if they had: active bleeding, or a bleeding diathesis; previous history of cerebrovascular event; use of clopidogrel, prasugrel or ticagrelor within 7 days of presentation; or haemodynamic instability.

The study participants received standard care for STEMI at our institution, comprising an oral loading dose of 300 mg aspirin administered in the community or after admission to hospital plus a loading dose of 60 mg prasugrel as soon as possible after admission in the emergency room or cathlab. At the start of PPCI, participants also received a 0.75 mg/kg bolus of bivalirudin followed by a 1.75 mg/kg/h infusion for the duration of the procedure. Use of unfractionated heparin was discouraged and participants were withdrawn if their management required use of a glycoprotein receptor inhibitor or continuation of the bivalirudin infusion after the PPCI.

### Platelet function tests

**Fig 1:**
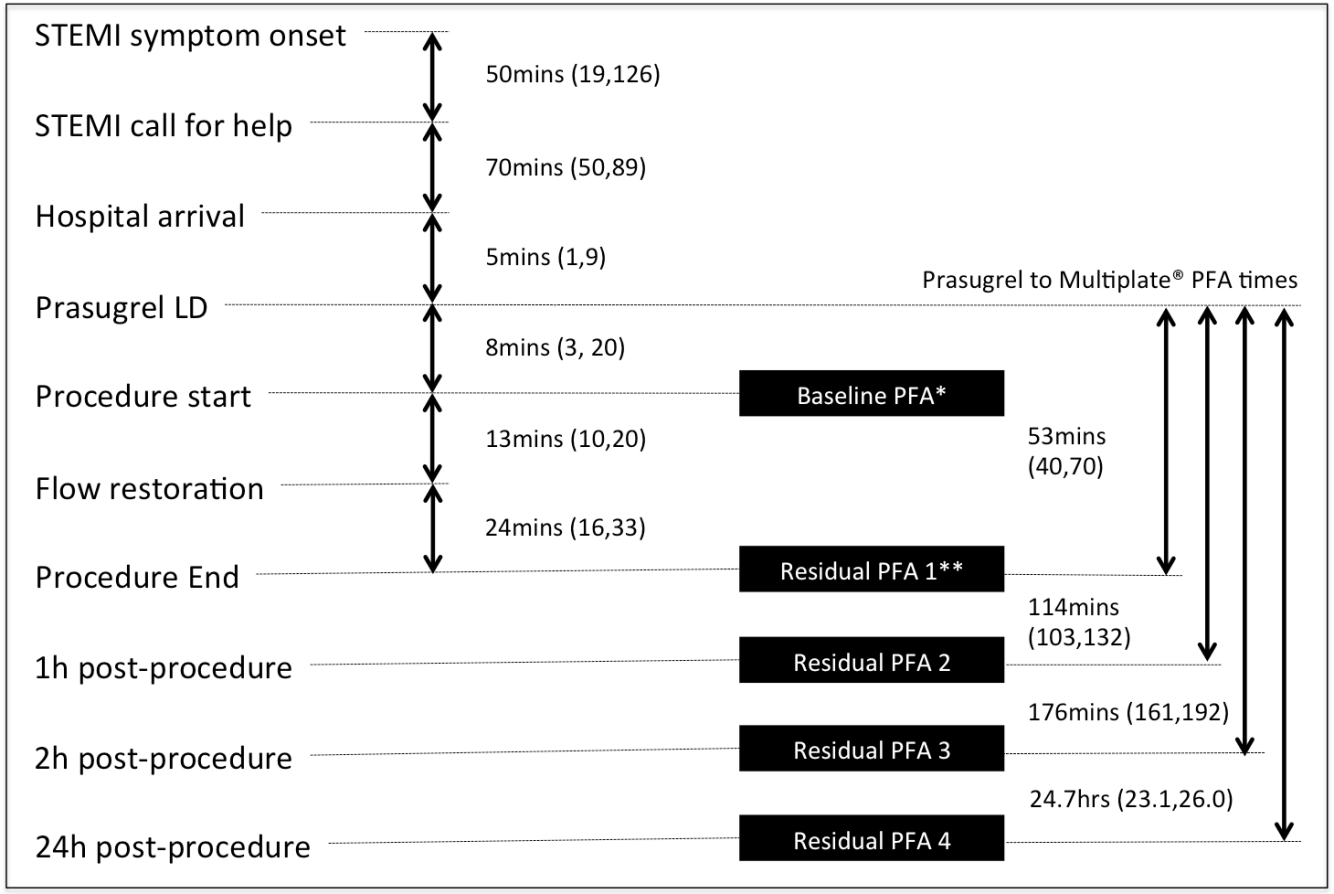
PINPOINT-PPCI study timeline. LD–loading dose, PFA–platelet function assessment. Times displayed are medians and IQRs. *The median baseline PFA time was 2 minutes after procedure start (IQR -1 to 6). **The median residual PFA1 time was 0 minutes post procedure (IQR 0 to 2). Missing data were as follows: 1 patient was missing flow time; 5 patients were missing baseline PFA times; 4 patients were missing residual PFA1 times; 8 patients were missing residual PFA2 times; 8 patients were missing residual PFA3 times; 17 patients were missing residual PFA4 times.

Venous blood samples for platelet function testing were collected into hirudin vacutainer tubes (Verum Diagnostica GmbH, Germany) on arrival at hospital, immediately after completion of the PPCI and 1, 2 and 24 hours post-procedure (Fig 1). At each time point, three platelet function assays were measured using whole blood electrical impedance aggregometry (Multiplate® analyzer, Roche Diagnostics International Ltd., Switzerland), namely ADP (ADP-test, Roche Diagnostics GmbH), arachidonic acid (ASPI-test, Roche Diagnostics GmbH) and thrombin receptor activating peptide (TRAP; TRAP-test, Roche Diagnostics GmbH). Additionally, the blood samples collected on arrival at hospital and at 24hours post-PPCI were also analysed to assess Thromboxane A_2_ receptor activity, using the synthetic Thromboxane A_2_ receptor agonist U46619. Platelet responses to test reagents were expressed as the area under the curve (AUC) of the change in electrical impedance in the first 6 minutes of analysis, presented in units (U; 1 U corresponds to 10 AU*min).

### Study outcomes

The primary outcome was the AUC for the response to the ADP-test. The secondary platelet function outcomes were the AUCs for the responses to ASPI-test, TRAP-test and U46619.

The secondary clinical outcomes were the incidence of major adverse cardiac events (MACE–a composite of target vessel revascularisation, non-fatal MI, and cardiac death), bleeding complications (using the trials in myocardial infarction (TIMI) major and minor bleeding criteria[11]) and ST (Academic research consortium (ARC) definition[12]) at 24 hours and 30 days after PPCI.

### Additional data collected

For each participant, we recorded the time of onset of symptoms, admission to hospital and the start and end times of the PPCI procedure. We also recorded the baseline characteristics of the participants, the time of administration of anti-thrombotic and other drugs, and procedural characteristics of the PPCI. We recorded data on several potential confounding factors that affect the characteristics of the index MI and might also have influenced door-to-balloon time and platelet reactivity on presentation. These were timing of symptoms/presentation relative to the time of arrival at hospital, age, diabetes, and prior long-term treatment with aspirin.

### Statistical methods

There were two key predictors of interest, namely door to end of procedure time and platelet activity at the time of arrival at hospital (baseline). We made the following assumptions to estimate the target sample size required to detect a statistically significant effect of key predictors of platelet function in a regression model: r^2^ = 0.25 for the full model; r^2^ = 0.2 for the model excluding the key predictors but with up to eight covariates; correlation of 0.5 between repeated measures; inclusion of two key predictors of interest in the model. Given these assumptions, a sample size of 108 was required to detect a gradient different from zero with 80% power and 5% significance (Bonferroni-corrected due to estimating the effects of two predictors).

Continuous data are presented as means and standard deviations (SD), or medians and interquartile ranges (IQRs) if distributions appear to be skewed. Categorical data are presented as numbers and percentages.

Analyses followed a pre-specified statistical analysis plan. The primary and secondary platelet function outcomes (ADP, ASPI, TRAP and U46619), measured at four time points after completion of PPCI, were analyzed using linear mixed model regression fitting the two key predictors and time as fixed effects regardless of significance. The interaction between the two key predictors was also included if it reached statistical significance at 10%, as were the interactions between each key predictor and time. A correlated participant-term random effect was included in each model to allow for repeated measures. In the pre-specified analysis plan, we decided not to apply any Bonferroni correction in the analyses.

Results are reported as effect estimates with 95% confidence intervals (CI). If the interaction with time was significant, estimates at each of the four time points are presented; otherwise estimates that are constant over time are presented. We adjusted for the potential confounding factors described above as fixed effects if they reached statistical significance at the 5% level. Model validity was checked using standard graphical methods and transformations were explored if a model fitted poorly. Outcomes analyzed on the logarithmic scale were transformed back to the original scale after analysis and results presented as geometric mean ratios (GMR).

The intention in the study protocol was to analyze the secondary safety outcomes (MACE, bleeding and ST) using logistic regression, with adjustments as stated above. However, due to insufficient numbers of events they are presented descriptively.

A post-hoc analysis of the platelet activity measurements by whether or not participants received morphine (or morphine plus anti-emetics) on hospital arrival is also reported, prompted by previous studies suggesting an association between opiate use and delayed P2Y12 antagonist platelet inhibition[4,13]. As above, these were analyzed using linear mixed model regression, with baseline and follow up platelet function as outcomes, and morphine (three-level categorical variable), time and the interaction between the two fitted as fixed effects. The interaction was only included if it was statistically significant at the 10% level.

## Results

One hundred and eight patients participated in the study. The mean age was 61.1years (SD 11.7) and 86/108 (79.6%) were male. Further characteristics of the participants are presented in Table 1.

**Table 1:** Baseline clinical and demographic characteristics.

		All patients (n = 108)	
**DEMOGRAPHICS**		**n**	**%**
Gender (male)		86/108	79.6%
Age (years) (mean, SD)		61.1	11.7
BMI (mean, SD) *		27.6	4.5
Heart rate (bpm) (mean, SD)		74.1	18.6
Systolic blood pressure (mean, SD) **		134.3	28.6
Diastolic blood pressure (mean, SD) **		81.7	19.8
Smoking status			
	Non-smoker	31/108	28.7%
	Smoker	45/108	41.7%
	Ex-smoker	32/108	29.6%
**MEDICAL HISTORY**		**n**	**%**
Diabetes		15/108	13.9%
	Diet/oral controlled	8/108	7.4%
	Insulin controlled	7/108	6.5%
Long term aspirin		21/108	19.4%
Family history of heart disease		54/108	50.0%
Peripheral vascular disease		3/108	2.8%
Hypercholesterolaemia		43/108	39.8%
Cerebrovascular disease		2/108	1.9%
Hypertension requiring treatment		38/108	35.2%
History of renal failure or dialysis		1/108	0.9%
Previous MI		7/108	6.5%
Previous PCI		5/108	4.6%
Previous CABG		2/108	1.9%

*1 patient missing data

**4 patients missing data

(CABG coronary artery bypass graft, MI myocardial infarction, PCI percutaneous coronary intervention, SD standard deviation)

The majority of cases were undertaken via the radial artery (101/108 patients, 93.5%) with one access site complication (0.9%), involving intense radial spasm requiring a general anesthetic to remove the guide catheter. Angiographic procedural success (residual stenosis <20%) was achieved in 105/107 cases (98.1%) with drug-eluting stents deployed in 56 cases (51.9%). Further procedural details are outlined in Table 2.

**Table 2:** Angiographic and procedural characteristics.

		All patients (n = 108)	
**PROCEDURAL TIMINGS**		**Median**	**IQR**
Onset of symptoms to arrival at BHI (mins)		135	(80.0, 207.5)
Door to end of procedure (mins)		55	(40.0, 70.0)
Total procedure time (mins)		38	(30.0, 51.5)
Call to balloon (flow) time* (mins)		101	(81.0, 123.0)
Door to balloon (flow) time* (mins)		27	(19.0, 40.0)
**PROCEDURAL CHARACTERISTICS**		**n**	**%**
Morphine on admission			
	Morphine	36/106	34.0%
	Morphine + anti-emetics	44/106	41.5%
	No morphine	26/106	24.5%
Bivalirudin infusion duration (mins) (median, IQR)*		30	(20.0, 42.0)
Unfractionated heparin		0/108	0.0%
GP2b/3a inhibitor used		0/108	0.0%
Radial access		101/108	93.5%
Femoral access §		13/108	12.0%
Thrombectomy*		84/107	78.5%
IABP inserted		1/108	0.9%
Access site complication ‡		1/108	0.9%
Stent type			
	BMS	51/108	47.2%
	DES	56/108	51.9%
	No stent	1/108	0.9%
Culprit vessel			
	Right coronary artery	53/108	49.1%
	Left anterior descending	32/108	29.6%
	Circumflex	22/108	20.4%
	Left main stem	0/108	0.0%
	Vein graft	1/108	0.9%
Procedural success*		105/107	98.1%

* 1 patient missing data

§ 6/13 = Failed radial, 7/13 default femoral

‡ Intense spasm right radial required anaesthetic

(BHI Bristol Heart Institute, BMS bare metal stent, DES drug-eluting stent, GP2b/3a inhibitor Glycoprotein 2b/3a receptor inhibitor, IABP intra-aortic balloon pump, IQR interquartile range)

One hundred and six patients (98.1%) received a 300 mg loading dose of aspirin, the majority (100/106) administered before admission to hospital (median time between aspirin loading and start of PPCI 61 minutes (IQR 41, 87)). One patient received a 150mg loading dose of aspirin and another received 900 mg. Twenty one participants (19.4%) presented on long term aspirin therapy. All participants received the loading dose of prasugrel at a median interval of 8 minutes (IQR 3, 20) after admission to hospital. The median duration of bivalirudin infusion was 30 minutes (IQR 20, 42). Further details of procedural and study timings are outlined in Fig 1.

### Primary outcome

**Fig 2:**
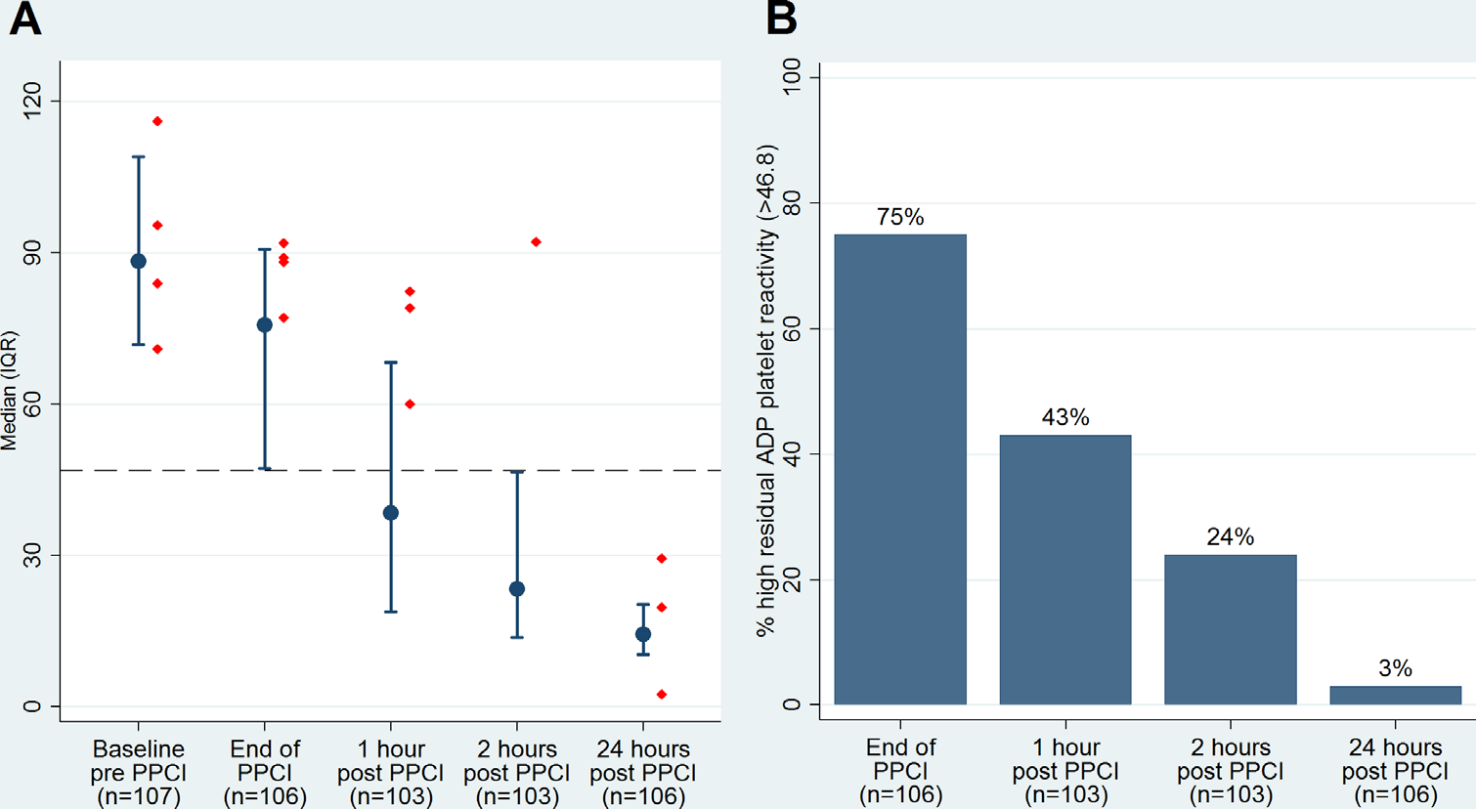
ADP platelet reactivity profile. Panel A—Median ADP receptor platelet activity profile in first 24 hours post-presentation with STEMI and treatment with PPCI. Red markers identify platelet response for the four acute ST patients (dotted line indicates the high residual platelet reactivity threshold of 46.8 U for ADP-test). Panel B–The profile of high residual platelet activity, in the first 24 hours, following administration of a 60 mg Prasugrel loading dose at the time of PPCI.

At admission to hospital, the median platelet ADP response was 88.3 U (IQR 71.8, 109.0; the manufacturer's reference interval for controls not receiving antiplatelet drugs is 57–113 U). Immediately after completion of PPCI the median ADP response was 75.7 U (IQR 47.2, 90.7), falling to 38.4 U (IQR 18.8, 68.3) 1 hour after PPCI, 23.4 U (IQR 13.7, 46.5) 2 hours after PPCI and 14.4 U (IQR 10.3, 20.3) 24 hours after PPCI (Fig 2A). A total of 80/106 (75%) participants displayed high residual platelet reactivity immediately after PPCI (HRPR; previously defined as ADP response >46.8 U[14]) indicating incomplete platelet P2Y12 blockade. This number reduced to 44/103 (43%) 1 hour after PPCI, 25/103 (24%) 2 hours after PPCI and 3/106 (3%) 24 hours after PPCI (Fig 2B).

**Fig 3:**
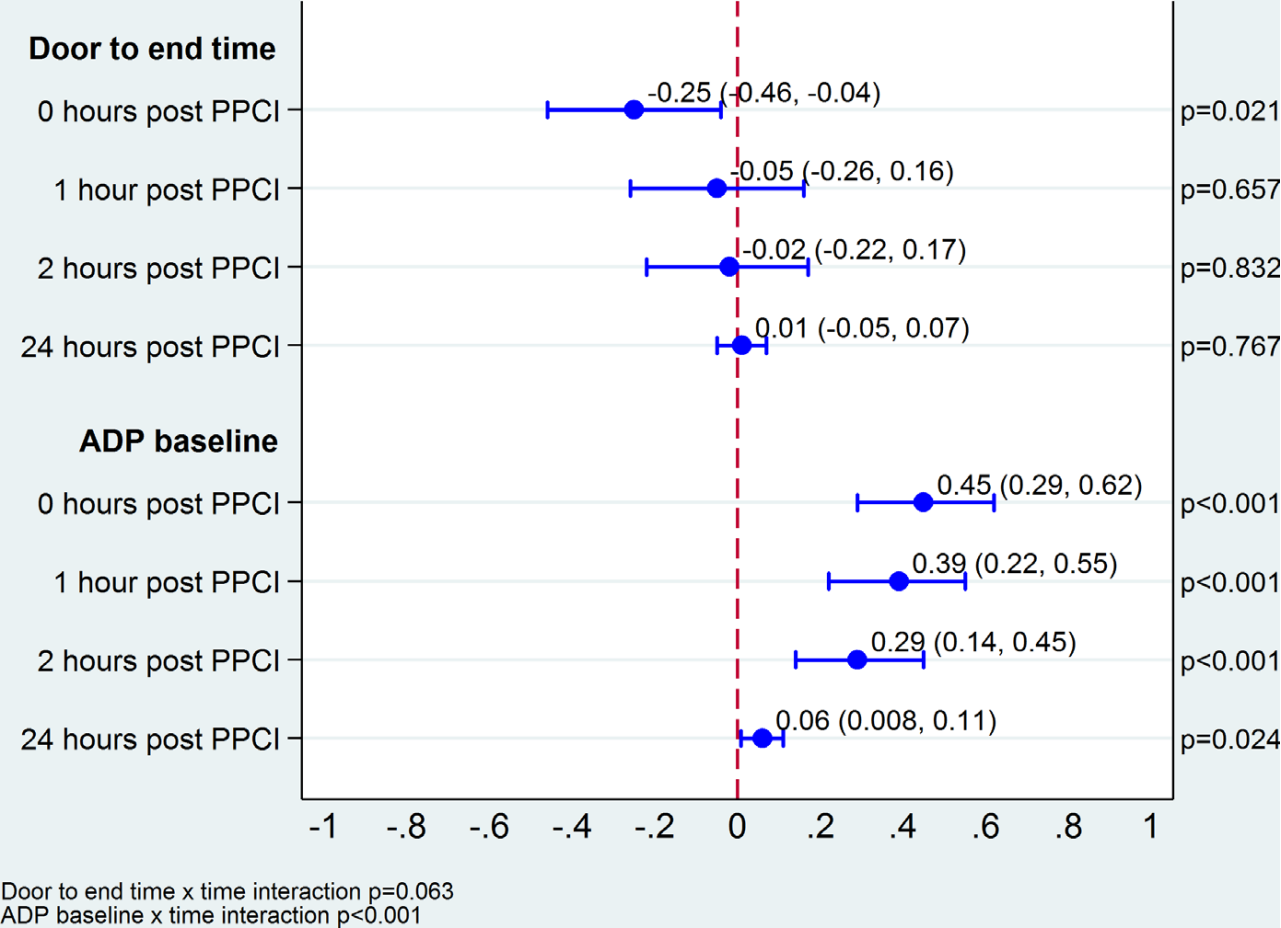
Effect of door to end of procedure time and baseline ADP platelet activity on ADP platelet function in the first 24 hours post-presentation with STEMI and treatment with PPCI.

Baseline ADP activity had a large effect on ADP immediately post intervention; for every unit increase in ADP response at admission, the ADP response increased on average by 0.45 U (95% CI 0.29 to 0.62), see Fig 3. The magnitude of this effect reduced substantially over time but was still apparent 24 hours after PPCI (0.06 U, 95% CI 0.008 to 0.11). Door to end of procedure time had a small but statistically significant effect on ADP immediately post procedure; for every minute increase in door to end time, ADP decreased by 0.25 U (95% CI -0.46 to -0.04), on average. This effect did not persist for the other three time points (Fig 3).

### Secondary outcomes

The median ASPI-test platelet response was 27.9 U (IQR 20.3, 53.2) at admission (manufacturer's reference interval for controls not receiving antiplatelet drugs 71–115 U) falling slowly and consistently over the subsequent time points to a median of 7.3 U (IQR 3.2, 13.0) at 24 hours post-PPCI. The platelet TRAP-test and U44619 responses showed similar kinetics (S2 Fig).

As baseline ASPI increased, post intervention ASPI on average increased, with this effect reducing in magnitude over time (immediately post procedure estimate 0.65, 95% CI 0.54 to 0.75, 24 hours post procedure estimate 0.06, 95% CI 0.02 to 0.10). Similarly, as baseline TRAP increased, post procedure TRAP on average increased; this effect persisted at all four time points. U44619 activity was only measured at baseline and 24hours post procedure. Results are presented as GMRs because the data were transformed. Baseline U44619 had a small but statistically significant effect on 24hour U44619 such that for every 10unit increase in baseline U44619, 24hour U44619 was on average 2.5% higher. Door to procedure end time had no effect at any time point after PPCI for the secondary outcomes ASPI-test, TRAP-test and U44619 (S3 Fig).

There were no associations of patient age, presence of diabetes, interval between the onset of symptoms and admission and previous longterm aspirin treatment with the platelet responses at the end of PPCI (data not shown).

### Adverse events / safety

During the 30 day follow-up period, five patients (4.6%) experienced MACE; four were acute ST and one was a sub-acute ST, all resulting in target vessel re-infarction and re-intervention. All four acute STs occurred within 2 hours of completion of procedure. Intravascular imaging confirmed that there had been a procedural complication in 2 of the 4 events (malapposition and distal stent edge disruption). The sub-acute ST occurred 18 days after the index PPCI, 5 days after stopping prasugrel in anticipation of coronary arterial bypass grafting for severe bystander disease. No significant bleeding was observed. Further to these pre-specified safety outcomes, there were 11 serious adverse events reported in nine patients. Two were during initial hospital stay and resulted in prolonged stay, and nine were after initial hospital discharge and resulted in readmissions. No patients died during the 30 day follow-up period. See S1 Table for details.

### Post-hoc analyses

**Fig 4:**
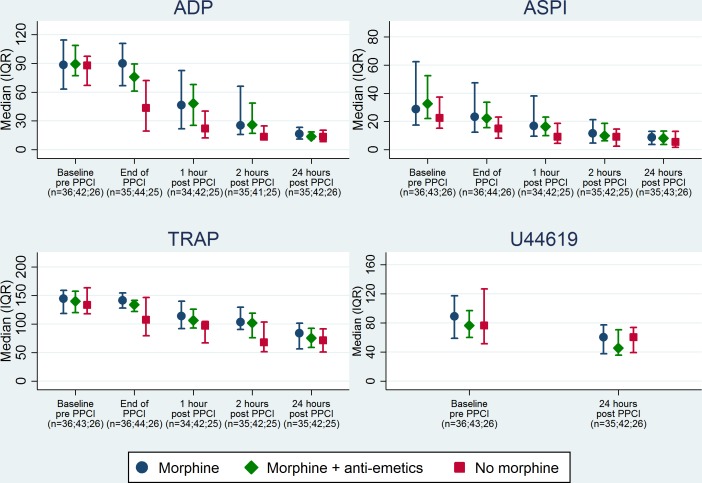
Influence of pre-procedural opiate and anti-emetic treatment on platelet function in the first 24 hours post-presentation with STEMI and treatment with PPCI. ADP: morphine effects pre-PPCI p = 0.56, end of PPCI p<0.001, 1 hour post-PPCI p = 0.035, 2 hours post-PPCI p = 0.007, 24hours post-PPCI p = 0.16; morphine x time interaction p<0.001. ASPI: morphine effect p = 0.67; morphine x time interaction p = 0.36. TRAP: morphine effect p = 0.08; morphine x time interaction p = 0.30. U44619: morphine effect p = 0.43; morphine x time interaction p = 0.82.

The influence of baseline morphine use on platelet activity was investigated in post-hoc analyses for all four platelet measures; 36/106 (34.0%) patients received morphine alone, 44/106 (41.5%) received morphine plus anti-emetics and 26/106(24.5%) patients received no morphine. The effect of morphine on ADP significantly differed across the five time points (morphine group by time interaction p<0.001). The strongest effect was observed at the end of PPCI (p<0.001); patients who received morphine before the procedure had higher levels of ADP (median 90.1 U, IQR 66.8–110.8, n = 35) compared to those who received morphine plus anti-emetics (median 75.9 U, IQR 61.1–89.4, n = 44) or those who did not receive morphine (median 43.5 U, IQR 19.3–72.1, n = 25). A significant morphine effect was also observed at 1 and 2 hours post-PPCI (p = 0.035 and p = 0.007, respectively); no morphine effect was observed at baseline or 24 hours post-PPCI (p = 0.56 and p = 0.16, respectively). Morphine was found to influence TRAP with borderline statistical significance (p = 0.08), although there was no evidence that this effect differed over time (morphine group by time interaction p = 0.30). No clear pattern was seen when investigating the effect of morphine on ASPI and U44619 (Fig 4).

## Discussion

This study describes the largest cohort of STEMI patients, treated acutely with prasugrel and bivalirudin, undergoing detailed platelet function profiling in the first 24 hours of treatment. We have demonstrated significant heterogeneity of baseline platelet activity. We have confirmed a relationship between baseline ADP activity and the post-procedural ADP activity measurements, following acute loading of prasugrel and a peri-procedural bivalirudin infusion. The same relationship was observed with assessment of thrombin receptor and arachidonic acid pathway platelet function.

A short door to end of procedure time resulted in a reduced immediate post-procedural efficacy of P2Y12 inhibition. However, we observed effective aspirin-related platelet inhibition in more than 75% of patients at the end of procedure, reflecting pre-hospital administration of the drug by ambulance staff and the excellent bioavailability of aspirin. In contrast, and consistent with previously published data[4,15], we observed relatively ineffective inhibition of the ADP receptor function by prasugrel, as judged by the proportion of patients with HRPR; 75% patients had HRPR at the end of procedure (53 minutes post-prasugrel LD), falling to 24% 2 hours post-procedure (176 minutes post-prasugrel LD). This differs markedly from the pharmacokinetic data achieved in healthy volunteers and stable patients but is unsurprising in view of the high baseline platelet activity and the rapid transit of patients through the cathlab (median prasugrel to procedure start time 8 minutes and median procedure time 38 minutes). The use of a double loading dose has been shown to enhance platelet inhibition with a significant reduction in patients with HRPR at 2 hours[16]. Alternative strategies to enhance platelet inhibition have been tested with other anti-platelet agents, including: pre-hospital drug administration[17], crushing of tablets[18] and use of an intravenous P2Y12 receptor inhibitor[19].

Large-scale clinical trials have highlighted a potential risk of acute ST relating to the abrupt cessation of bivalirudin at the end of the PPCI procedure[6,9]. Our study used the same protocol of bivalirudin administration and we observed a high rate of acute ST (3.7%), all occurring within the first 2 hours post-procedure. It would seem likely that the HRPR observed within our study cohort, combined with the rapid reversal of the effect of bivalirudin following cessation of the drug (half-life approximately 25minutes[20]) conspire to heighten the risk of acute ST.

In lower risk cohorts, multiplate platelet function testing has successfully been used to identify patients at risk of ST[21]. Similarly, adverse events have been associated with HRPR detected in ACS patients loaded with prasugrel[15]. However, subsequent large multicentre trials attempting to ‘tailor’ therapy with adjustment of antiplatelet therapy according to HRPR have failed to demonstrate benefit[22–25]. Our intensive profiling of platelet function during the first 24 hours of treatment for STEMI offers potential insights into the aetiology of early adverse events. Acute ST was observed within 1–2 hours of the procedure (cessation of bivalirudin). However, it does not appear that the baseline or immediate post-procedural platelet function test could be used to predict ST or facilitate individualized therapy. The baseline ADP platelet activity in the 4 patients suffering acute ST was well distributed (Fig 2), and 75% of the entire cohort failed to achieve effective ADP inhibition at the time of stopping bivalirudin.

Post-hoc analysis of our data supports the existence of a relationship between opiate use and impaired platelet inhibition[4]. Pooled data from a series of STEMI trials involving platelet function assessment (predominantly VerifyNow)[26] have confirmed the association between opiate use and delayed platelet inhibition following ticagrelor or prasugrel loading, even when adjusting for vomiting. Our use of multiplate platelet function analysis extends the understanding of this relationship, through simultaneous assessment of platelet ADP, arachidonic pathway, and thrombin receptor function. Baseline ADP activity did not appear to be influenced by the administration of opiates (Fig 4). However, at subsequent time points, we observed delayed ADP platelet inhibition in the presence of opiate, with some reduction of the effect when an anti-emetic was co-administered. This finding strengthens the hypothesis that the opiate effect on platelet function relates to a combination of increased vomiting and reduced drug absorption with a less potent oral P2Y12 inhibitor, clopidogrel[13]. Interestingly, ASPI platelet function was unaffected by opiate administration. In the majority, aspirin was co-administered with the opiate by a paramedic (aspirin loading occurred 61minutes (IQR 41 to 88) before commencement of PPCI) and the excellent bioavailability of the drug is likely to have facilitated effective platelet inhibition. Similarly, no difference in TRAP activity was observed between patients receiving opiate and those naïve of the drug.

### Study Limitations

Several important limitations to the study design merit discussion. Most important was the need to exclude patients requiring additional treatment with anti-thrombotic/glycoprotein inhibitor therapy. This exclusion is the reason for the low MACE and bleeding rates observed but does not change the observations about platelet function among the eligible participants. Additionally, the requirement for platelet function assessment at multiple time points limited recruitment to periods when research staff were available to undertake analysis. Consequently, there was a selection bias to patients presenting during working hours but we know of no reason why the findings should vary by time of presentation.

Despite a number of patients experiencing acute ST, a full platelet profile across all 5 timepoints was not possible for these patients due to the need for emergency intervention and the subsequent use of intravenous glycoprotein antagonists. Additionally, the study was undertaken in a single centre and the relatively small sample size limited the interpretation of observed clinical endpoints.

The interaction between the increasing platelet inhibition following administration of a pre-procedural prasugrel loading dose and the waning anti-thrombotic effect of bivalirudin, following cessation of the infusion, at the end of the PPCI procedure, is likely to contribute to the occurrence of acute ST. Unfortunately, despite a measurable effect of bivalirudin on platelet aggregation using Multiplate[27], our analysis is unable to distinguish between the effects of the two drugs administered during the acute intervention.

Parameters of interest were fitted in multiple regression models and, when drafting the analysis plan, we judged that a Bonferroni correction would be too conservative. We acknowledge that multiplicity of comparisons may be an issue but, nevertheless, pre-specified that we would not adjust p-values. Consistency of findings is an important consideration when judging causality[28] and we have no information on which to base an adjustment or choice of a criterion for assigning significance that would be less conservative than Bonferroni. In these circumstances, we believe that (a) we should not depart from our analysis plan and (b) applying one or other method would represent an arbitrary decision, which would not aid interpretation of the findings.

## Conclusions

The acute nature of STEMI presentation, requiring immediate invasive intervention, combined with high baseline platelet activity and a tendency to thrombosis poses major challenges. The balance of thrombosis and bleeding is delicate. We have confirmed significant variability in baseline platelet ADP function with a persisting effect on ADP activity in the first 24 hours, despite early oral antiplatelet administration. The pursuit of rapid mechanical recanalization of the occluded artery further impacts on immediate post-procedural ADP receptor inhibition, with a persisting high rate (24.3%) of HRPR 2 hours following PPCI. Additionally, administration of opiates in the acute setting appears to further delay inhibition of platelet ADP activity. The combination of prasugrel and bivalirudin provides an excellent safety profile, with regards to bleeding, but is associated with a high rate of acute ST, if bivalirudin is stopped at the end of the procedure.

An optimal pharmacological strategy for PPCI is yet to be found but our data supports further evaluation of methods to accelerate platelet inhibition or prolong thrombin inhibition.

## Supporting Information

S1 STROBE Checklist (DOCX)

S1 Fig Consort flow diagram. (DOCX)

S2 Fig The median platelet activity profiles in the first 24hours post-PPCI. Median platelet activity profiles for arachidonic acid pathway (ASPI–Panel A), thrombin receptor (TRAP–Panel B) and thromboxane A_2_ receptor (U44619 –Panel C) function in the first 24 hours post-presentation with STEMI and treatment with PPCI. Red markers identify platelet response for the four acute ST patients (dotted line indicates the high residual platelet reactivity threshold of 40 U for ASPItest)
(TIF)

S3 Fig The effect of door to end of procedure time and baseline platelet activity on secondary outcome measures. Effect of door to end of procedure time and baseline platelet activity on arachidonic acid pathway (ASPI–Panel A), thrombin receptor (TRAP–Panel B) and thromboxane A_2_ receptor (U44619 –Panel C) platelet function in the first 24 hours post-presentation with ST-elevation myocardial infarction and treatment with primary percutaneous coronary intervention (PPCI). ASPI: door to end time x time interaction p = 0.394, ASPI baseline x time interaction p<0.01. TRAP: door to end time x time interaction p = 0.394, TRAP baseline x time interaction p = 0.083. U44619: Only collected at baseline and 24 hours post-PPCI.
(TIF)

S1 Table Serious Adverse Events. (DOCX)

S1 Text PINPOINT-PPCI Trial Protocol. (DOC)
