# Supplementary material for: A Study of Platelet Inhibition, Using a ‘Point of Care’ Platelet Function Test, following Primary Percutaneous Coronary Intervention for ST-Elevation Myocardial Infarction [PINPOINT-PPCI]
Source: PLoS One. 2015 Dec 16;10(12):e0144984. doi: 10.1371/journal.pone.0144984 (PMC4682629; doi:10.1371/journal.pone.0144984)
Supplement: S1 Fig — (DOCX) [file pone.0144984.s002.docx]

Figure Legend

Consort Flow Diagram for the PINPOINT-PPCI study

**Assessed for eligibility (n=139)**

**Patients excluded (n=31)**

**Ineligible (n=1):**

*Current Clopidogrel therapy*

**Did not consent (n=1):**

*Reason unknown*

**Other (n=29)**

*Reasons unknown*

**Withdrawals (n=0)**

**Consented to participate (n=108)**

**No post-procedure samples obtained (n=0)**

**Provided at least one post-procedure blood sample and included in analysis population (n=108)**

**Withdrawals post-hospital discharge (n=0)**

**Data for 30-day assessment not available (n=0)**

**30-day follow-up data collected and included in analysis of secondary outcomes (n=108)**
