# Supplementary figures and images for: A Study of Platelet Inhibition, Using a ‘Point of Care’ Platelet Function Test, following Primary Percutaneous Coronary Intervention for ST-Elevation Myocardial Infarction [PINPOINT-PPCI]
Source: PLoS One. 2015 Dec 16;10(12):e0144984. doi: 10.1371/journal.pone.0144984 (PMC4682629; doi:10.1371/journal.pone.0144984)

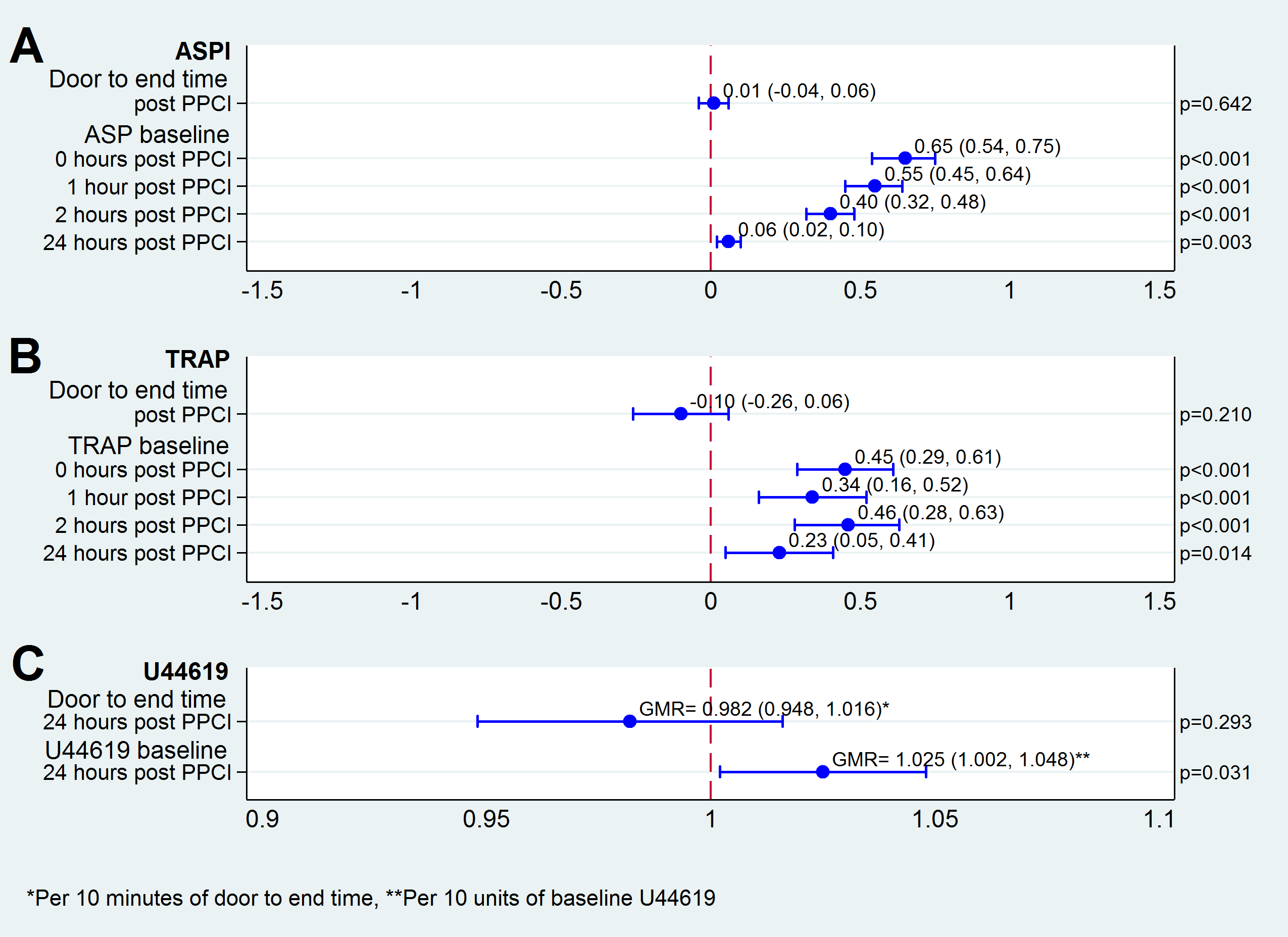

Supplement: S2 Fig — Median platelet activity profiles for arachidonic acid pathway (ASPI–Panel A), thrombin receptor (TRAP–Panel B) and thromboxane A2 receptor (U44619 –Panel C) function in the first 24 hours post-presentation with STEMI and treatment with PPCI. Red markers identify platelet response for the four acute ST patients (dotted line indicates the high residual platelet reactivity threshold of 40 U for ASPItest) (TIF) [file pone.0144984.s003.tif]

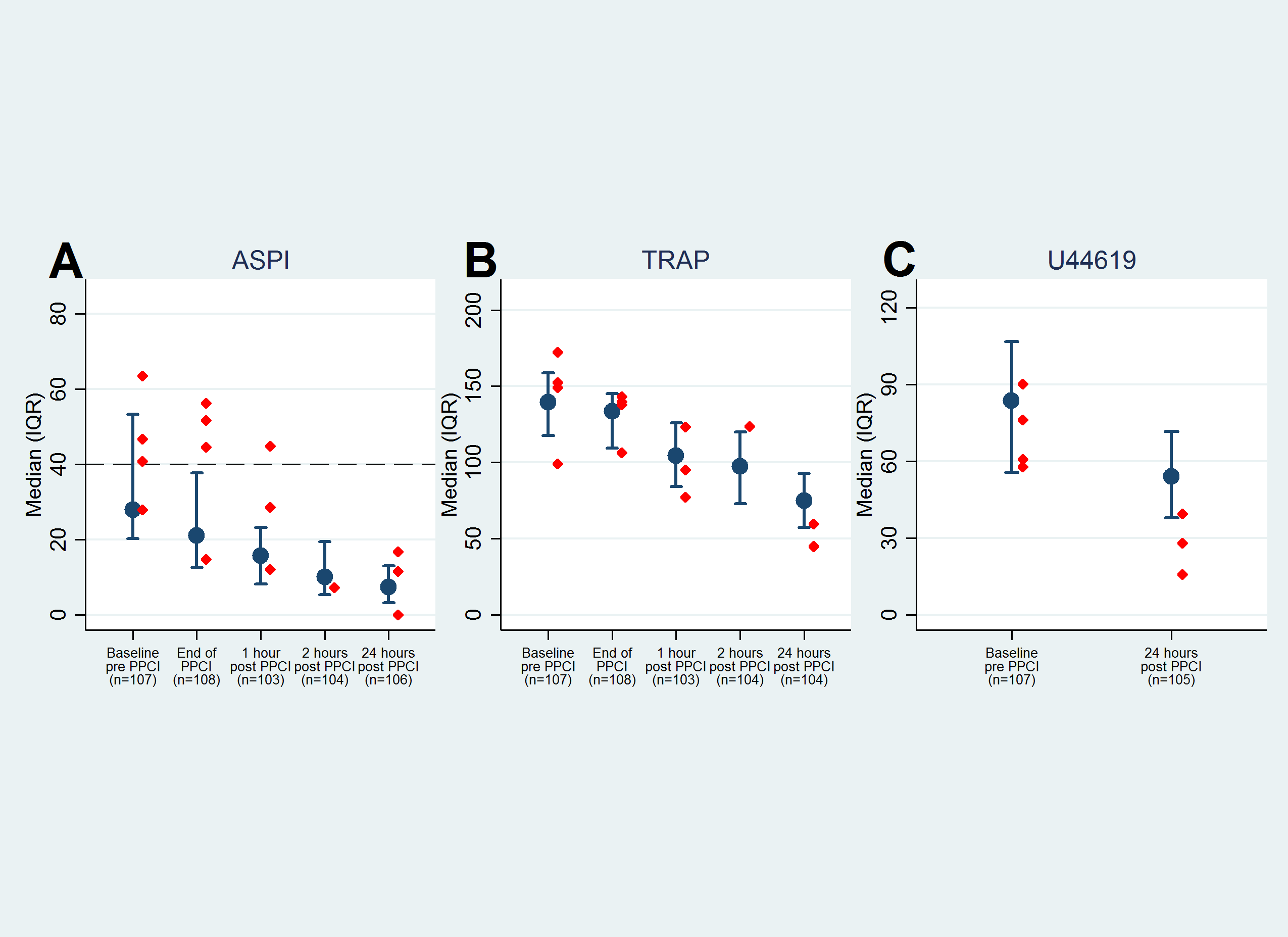

Supplement: S3 Fig — Effect of door to end of procedure time and baseline platelet activity on arachidonic acid pathway (ASPI–Panel A), thrombin receptor (TRAP–Panel B) and thromboxane A2 receptor (U44619 –Panel C) platelet function in the first 24 hours post-presentation with ST-elevation myocardial infarction and treatment with primary percutaneous coronary intervention (PPCI). ASPI: door to end time x time interaction p = 0.394, ASPI baseline x time interaction p<0.01. TRAP: door to end time x time interaction p = 0.394, TRAP baseline x time interaction p = 0.083. U44619: Only collected at baseline and 24 hours post-PPCI. (TIF) [file pone.0144984.s004.tif]
