# Supplementary material for: A Study of Platelet Inhibition, Using a ‘Point of Care’ Platelet Function Test, following Primary Percutaneous Coronary Intervention for ST-Elevation Myocardial Infarction [PINPOINT-PPCI]
Source: PLoS One. 2015 Dec 16;10(12):e0144984. doi: 10.1371/journal.pone.0144984 (PMC4682629; doi:10.1371/journal.pone.0144984)
Supplement: S1 Table — (DOCX) [file pone.0144984.s005.docx]

|  | **All patients (n=108)** | |
| --- | --- | --- |
| **SAEs** | **Events/ patients** | **%** |
| **Any SAE** | 11/9 | 8.3% |
| **Prolonged admissions:** |  |  |
| Pneumonia, VT defibrillator implant, renal impairment | 1/1 | 0.9% |
| Valvuloplasty | 1/1 | 0.9% |
| **Readmissions:** |  |  |
| Post MI inflammatory syndrome | 1/1 | 0.9% |
| Dizziness | 1/1 | 0.9% |
| Fatigue and weakness | 1/1 | 0.9% |
| Pre-syncopal episode | 1/1 | 0.9% |
| CABG surgery | 1/1 | 0.9% |
| Chest pain, no revascularisation needed. | 1/1 | 0.9% |
| Left upper arm weakness and erratic heart beat | 1/1 | 0.9% |
| Atrial fibrillation (discharged same day) | 2/1 | 0.9% |
