# Supplementary material for: A Study of Platelet Inhibition, Using a ‘Point of Care’ Platelet Function Test, following Primary Percutaneous Coronary Intervention for ST-Elevation Myocardial Infarction [PINPOINT-PPCI]
Source: PLoS One. 2015 Dec 16;10(12):e0144984. doi: 10.1371/journal.pone.0144984 (PMC4682629; doi:10.1371/journal.pone.0144984)
Supplement: S1 Text — (DOC) [file pone.0144984.s006.doc]

**Short project title:** Point of care platelet activity measurement in primary PCI

**Project Title:** A study of platelet inhibition, using a ‘point of care’ platelet function test, following primary percutaneous coronary intervention for ST elevation myocardial infarction (PINPOINT-PPCI)

**REC reference number: 10/H0106/87**

**R&D reference number: CS/2010/3568**

**Funding reference: Above & Beyond Project 353**

**Details of Sponsor**

University Hospitals Bristol NHS Foundation Trust

Research & Development Department

Level 3 – Upper Maudlin St.

Bristol BS2 8AE

Tel: 0117 342 0233

Fax: 0117 342 0329

**Chief Investigators & Research Team Contact Details**

Dr Thomas Johnson

Cardiologist

University Hospitals Bristol NHS Foundation Trust

Bristol Heart Institute

Tel: 0117 342 6567

E-mail: [tom.johnson@uhbristol.nhs.uk](mailto:tom.johnson@uhbristol.nhs.uk)

Dr Andreas Baumbach

Consultant Cardiologist & Reader

University Hospitals Bristol NHS Foundation Trust

Bristol Heart Institute

Tel: 0117 342 6631

E-mail: [andreas.baumbach@uhbristol.nhs.uk](mailto:andreas.baumbach@uhbristol.nhs.uk)

Dr Andrew Mumford

Consultant Haematologist & Senior Lecturer

University Hospitals Bristol NHS Foundation Trust

E-mail: [a.mumford@bristol.ac.uk](mailto:a.mumford@bristol.ac.uk)

Professor Barney Reeves

Professorial Research Fellow in Health Services Research

Bristol Heart Institute

E-mail: Barney.Reeves@bristol.ac.uk

# Table of Contents

[1 Table of Contents 2](#__RefHeading___Toc270422548)

[2 Glossary 3](#__RefHeading___Toc270422549)

[3 Definitions 3](#__RefHeading___Toc270422550)

[4 Lay Summary 4](#__RefHeading___Toc270422551)

[5 Background 4](#__RefHeading___Toc270422552)

[6 Hypothesis and Study Objectives 5](#__RefHeading___Toc270422553)

[7 Plan of Investigation 7](#__RefHeading___Toc270422554)

[7.1 Study Schema 7](#__RefHeading___Toc270422555)

[7.2 Study Design 7](#__RefHeading___Toc270422556)

[7.3 Outcomes 8](#__RefHeading___Toc270422557)

[7.3.1 Primary Outcomes 8](#__RefHeading___Toc270422558)

[7.3.2 Secondary Outcomes 8](#__RefHeading___Toc270422561)

[7.3.3 Confounding variables 8](#__RefHeading___Toc270422562)

[7.4 Study Population 8](#__RefHeading___Toc270422563)

[7.4.1 Inclusion Criteria 8](#__RefHeading___Toc270422564)

[7.4.2 Exclusion Criteria 8](#__RefHeading___Toc270422565)

[7.5 Sample size and justification 9](#__RefHeading___Toc270422566)

[7.6 Analysis plan 9](#__RefHeading___Toc270422567)

[8 Study Procedures 9](#__RefHeading___Toc270422568)

[8.1 Patient Presentation 9](#__RefHeading___Toc270422569)

[8.2 Standard Treatment of Patients 9](#__RefHeading___Toc270422570)

[8.2.1 Pre- & Peri-Procedural Care 9](#__RefHeading___Toc270422571)

[8.2.2 Post-procedural Inpatient Care 10](#__RefHeading___Toc270422572)

[8.2.3 Follow-up Care 10](#__RefHeading___Toc270422574)

[8.3 Research Procedures 10](#__RefHeading___Toc270422575)

[8.3.1 In-patient Procedures 10](#__RefHeading___Toc270422576)

[8.3.2 Follow-up Visits 11](#__RefHeading___Toc270422577)

[8.4 Duration of Study 11](#__RefHeading___Toc270422578)

[8.5 Data Collection 11](#__RefHeading___Toc270422579)

[8.6 Source Data 11](#__RefHeading___Toc270422580)

[8.7 Screening & Eligibility Assessment 11](#__RefHeading___Toc270422581)

[8.8 Planned recruitment rate 11](#__RefHeading___Toc270422582)

[8.9 Discontinuation/Withdrawal of Participants from Study 11](#__RefHeading___Toc270422583)

[9 Analyses 11](#__RefHeading___Toc270422584)

[10 Project Management 12](#__RefHeading___Toc270422585)

[11 Safety reporting 12](#__RefHeading___Toc270422586)

[12 Ethical considerations 12](#__RefHeading___Toc270422587)

[12.1 Ethical review 12](#__RefHeading___Toc270422588)

[12.2 Obtaining informed consent from participants 12](#__RefHeading___Toc270422589)

[13 Research governance 12](#__RefHeading___Toc270422590)

[13.1 NHS approval 13](#__RefHeading___Toc270422591)

[13.2 Investigators' responsibilities 13](#__RefHeading___Toc270422592)

[13.3 Monitoring by sponsor 13](#__RefHeading___Toc270422593)

[13.4 Indemnity 13](#__RefHeading___Toc270422594)

[14 Data protection and patient confidentiality 13](#__RefHeading___Toc270422595)

[14.1 Data handling 13](#__RefHeading___Toc270422596)

[14.2 Data storage 13](#__RefHeading___Toc270422597)

[14.3 Data sharing 13](#__RefHeading___Toc270422598)

[15 Dissemination of findings 14](#__RefHeading___Toc270422599)

[16 References 14](#__RefHeading___Toc270422600)

# Glossary

STEMI ST elevation myocardial infarction

MEA Multiple electrode analyzer

ECG Electrocardiogram

PPCI Primary percutaneous coronary intervention

PCI Percutaneous coronary intervention

CRF Case report form

ADP Adenosine di-phosphate

BHI Bristol Heart Institute

UFH Unfractionated heparin

GPI Glycoprotein 2b/3a Receptor Inhibitor

MACE Major adverse cardiac events

ASPI Arachidonic acid activation assay

AUC Area under curve

TIMI Trials in myocardial infarction

P2Y12 Specific ADP platelet receptor

A&E Accident and emergency

UHBristol University Hospitals Bristol NHS Foundation Trust

CRP C-reactive protein

eGFR Estimated glomerular filtration rate

CCU Coronary Care Unit

PIS Patient information sheet

CTEU Clinical Trials and Evaluation Unit

ECG Electrocardiogram

ARC Academic research consortium

GUSTO Global Use of Strategies to Open Coronary Arteries

# Definitions

STEMI Determined by a 12-lead ECG at the time of presentation with/without chestpain demonstrating either 1mm ST elevation in two contiguous leads or new-onset left bundle branch block.

Low platelet response Determined by comparison of ADPtest result from multiplate analyzer against a reference population of patients receiving aspirin and a P2Y12 receptor inhibitor, defined by Sibbing et al (ref). The lowest quintile (20%) of the population were identified to have significantly increased risk of adverse thrombotic events (i.e. stent thrombosis).

MACE A composite of target vessel and target lesion revascularization, cardiac death, and non-fatal myocardial infarction.

Stent thrombosis Using the Academic Research Consortium (ARC) definition(1) events are defined as definite, probable or possible and early (0-30days), late (31-360days) or very late (>360days). Definite stent thrombosis must be confirmed by angiographic or autopsy evidence of thrombosis/vessel occlusion. Probable stent thrombosis is defined as unexplained death within 30days of the index procedure, or evidence of acute myocardial infarction involving the target vessel territory. Possible stent thrombosis is classified as all unexplained death occurring at least 30days from the procedure.

Bleeding Using the GUSTO bleeding score(2) where events are classified as severe (deadly bleeding; intracerebral bleeding or substantial hemodynamic compromise requiring treatment), moderate (requiring transfusion) or mild (bleeding not requiring transfusion or causing hemodynamic compromise).

# Lay Summary

Heart attacks are increasingly being treated with immediate re-opening and placement of a metal-mesh tube (stent) in the blocked heart artery. In addition to scaffolding the artery with a stent, drugs are used to thin the patient’s blood. We have recently changed the drugs given to patients presenting to the Bristol Heart Institute with heart attacks. The drugs we have selected are potent blood thinners and have been shown to act very fast.

In this study we want to measure the level of blood thinning achieved at the end of the stenting procedure. We believe that there may be a variable response to the blood thinning drugs and patients with a low response may be at increased risk of blood clotting problems. We will describe the relationship between the measure of blood thinning at the end of the procedure with the time given for the drug to take effect and ultimately with complications developed within the first 30 days following the stenting procedure.

# Background

Optimal treatment of acute ST-elevation myocardial infarction (STEMI) involves rapid diagnosis, and transfer to a percutaneous coronary intervention (PCI)-capable cardiac centre for immediate mechanical revascularisation (3).

The primary-PCI (PPCI) service in Bristol is co-ordinated by the Bristol Heart Institute (BHI). Following a recent expansion of our catchment, incorporates the Gloucestershire, Avon & Wiltshire territories of the local network, we anticipate over 600 PPCI cases in the next year. We are achieving excellent results and our purpose-built unit allows rapid transfer of patients to the catheterisation laboratory with a median ‘door to balloon time’ of 39minutes, far exceeding the national average (median time 50minutes) (4).

Platelet activation occurs early in the cascade of events leading to coronary arterial occlusion and STEMI. Therefore anti-platelet therapy is fundamental to successful re-canalisation and subsequent reperfusion of the myocardium. In light of the ‘time-critical’ nature of STEMI treatment, current anti-thrombotic therapy involves use of both intravenous and oral agents to confer both immediate and long-term anti-thrombotic effects.

Significant advances in the inhibition of platelet activity have occurred in recent years and current data supports the use of increasingly potent anti-thrombotic agents and platelet inhibitors to limit the risk of future cardiac events including mortality. However, increasingly potent and advanced pharmacotherapy comes at a financial cost and at a cost of an increased risk of complications, particularly bleeding. A balance has to be achieved between effective suppression of thrombosis, prevention of significant bleeding and cost.

We have recently amended our anti-thrombotic protocol in the treatment of STEMI - converting from using un-fractionated heparin (UFH) with glycoprotein 2b/3a receptor inhibitors (GPI) to bivalirudin monotherapy in the catheter laboratory, with additional substitution of prasugrel for clopidogrel. Prasugrel is administered to patients on arrival at the Bristol Heart Institute prior to undertaking angiography and PPCI. Use of bivalirudin has been shown to offer equivalent benefit in terms of limiting major adverse cardiac events (MACE), with a reduction in cardiac mortality and bleeding vs UFH plus GPI (5). Prasugrel is a potent inhibitor of the platelet ADP receptor P2Y12. It was tested against clopidogrel in the TRITON-TIMI 38 trial and demonstrated a significant reduction in a composite of cardiac mortality, non-fatal myocardial infarction and non-fatal stroke in the treatment of STEMI (6).

The use of prasugrel and bivalirudin in the acute treatment of STEMI offers excellent anti-platelet and anti-thrombotic effect (5,6). The combination of prasugrel with bivalirudin should confer increased protection against early thrombotic events, as prasugrel has been demonstrated to offer faster inhibition of platelet activity vs clopidogrel (7). However, it should be noted that the HORIZONS-AMI trial, assessing bivalirudin vs. UFH/GPI, revealed an increased rate of stent thrombosis at 24hours with bivalirudin monotherapy. Reassuringly, at 30 days the stent thrombosis rate was not statistically different in both treatment groups (8). This transient early negative outcome with bivalirudin most likely relates to the pharmacokinetics of the drug, and the protocol of administration. Bivalirudin is administered intravenously, with an initial bolus (0.75mg/kg) and then an infusion (1.75mg/kg/h) until the completion of the angiography/PCI procedure. Bivalirudin has a half-life of ≈25minutes and, therefore, thrombin activity is restored fairly rapid when the infusion stops. The increase in acute stent thrombosis may signal a gap in anti-thrombotic protection arising from the waning anti-thrombin effect of bivalirudin and the increasing anti-platelet effect of prasugrel on the P2Y12 receptor inhibitor.

The newly amended BHI anti-thrombotic protocol assumes that all STEMI patients will achieve adequate platelet inhibition with prasugrel by the time bivalirudin is stopped, immediately after treatment with a coronary stent. The validation of this assumption in’real’ STEMI patients has not been tested. This is the aim of the study.

# Hypothesis and Study Objectives

Minimising the time from STEMI symptom onset to definitive treatment, with mechanical revascularisation, remains a major focus of attention in optimising the PPCI service. We hypothesise that continued reductions in the “door to balloon” time (time from hospital admission to achieving an open artery in the catheter laboratory) may result in some patients undergoing PPCI with incomplete P2Y12 blockade, leading to an increase in risk of early thrombotic events. We speculate that the rapid restoration of coronary flow achieved with PPCI may offer inadequate time to achieve platelet inhibition with prasugrel, prior to the loss of the anti-thrombin effect of bivalirudin.

The primary objective of the study is to describe variation in platelet activity at the end of the PPCI and to quantify the relationship between “door to balloon time” and platelet function in the first 24 hours after completing the PPCI.

The secondary objective of the study is to test the hypothesis that patients with high platelet activity at the time of arrival at hospital (baseline) achieve less platelet inhibition in the first 24 hours following commencement of anti-thrombotic and anti-platelet therapy.

# Plan of Investigation

## Study Schema

STEMI

BHI

Catheter Lab for PPCI

Bivalirudin

Prasugrel 60mg bolus

Prasugrel 10mg daily

24hours

& 30day

Clinical

Follow-up

Multiplate Platelet Function Assessment

**TIME 0**

Admission

**TIME 1**

Immediate

Post PCI

**TIME 2**

1hours

Post PCI

**TIME 3**

2hours

Post PCI

**TIME 4**

24hours

Post PCI

## Study Design

This is a prospective observational study.

## Outcomes

### Primary Outcomes

The primary outcome for study is the platelet function assessment measured in peripheral ‘whole’ blood. Functional assessment of ADP receptor, arachidonic acid pathway, and thrombin related platelet activation will be measured using a multiple electrode analyser (MEA – Multiplate platelet function analysis).

Platelet function (measured in peripheral ‘whole’ blood, see above) will be assessed on arrival at hospital, at completion of the PPCI, 1, 2 and 24 hours after the completion of the PPCI. These measurements will allow the study to test how the profile of platelet function changes over the period of time in which the effect of bivalirudin wanes and prasugrel increases, by assessing the interaction between door-to-balloon time and time-after-completion of PPCI.

### Secondary Outcomes

Incidence of adverse clinical events at 24hours and 30days, including:

- Major adverse cardiac events (MACE) – a composite of target vessel revascularisation, target lesion revascularisation, non-fatal myocardial infarction, and cardiac death
- Bleeding complications – using TIMI major and minor bleeding criteria
- Stent thrombosis (ARC definition, see Section 3)

### Confounding variables

We will measure and record confounding variables associated with the characteristics of the index MI and platelet reactivity. Variables will include: timing of symptoms/presentation relative to initiation of treatment, the maximum CKMB/Troponin T, age, gender, co-existent diabetes, concomitant treatments, high sensitivity CRP and WCC.

## Study Population

We propose prospective recruitment of 108 STEMI patients undergoing PPCI at the BHI, receiving standard care with prasugrel loading on admission and subsequent bivalirudin bolus and infusion therapy peri-procedurally. Assuming a 60% recruitment rate, we would anticipate completing recruitment within 6 months.

### Inclusion Criteria

A participant may enter the study if ALL of the following apply:

- Admitted with acute STEMI
- Treated with PPCI at the time of index procedure

### Exclusion Criteria

A participant may not enter the study if ANY of the following apply:

1. Unable to take prasugrel
2. Unable to take bivalirudin
3. Haemodynamic instability/cardiogenic shock
4. Current treatment with Clopidogrel or Prasugrel

## Sample size and justification

The primary analysis will be a mixed model regression (see below). Calculation of the sample size needed to detect a statistically significant effect of a key predictors in such analyses require the following assumptions to be defined (parameter = estimate):

r-squared for full model = 0.25

r-squared for reduced model (excluding key predictor) = 0.20

number of predictors in full model = 8

number of predictors being tested = 1

Because two key predictors will be tested (see below), a significance level of 0.025 will be adopted (Bonferroni correction) giving a nominal hypothesis test at p<0.05 for each predictor. For 80% power, the above assumptions require a target sample size of 135. This target sample size needs to be further adjusted by the relative efficiency of the analysis, i.e. to take into account the three repeated measures. Estimating the relative efficiency requires further assumptions about the correlations between the repeated measures; for correlations ranging from 0.5 to 0.8, the relative efficiency varies from 1.25 to 1.50. Taking the lower estimate of relative efficiency gives a final target sample size of 108 (i.e. 135/1.25).

## Analysis plan

The primary analysis will be a mixed model regression to take into account the three repeated measures of platelet function at 1, 2 and 24 hours after completion of PPCI. Platelet function on arrival at the hospital, and platelet function after completion of PPCI, are not expected to be influenced by door-to-balloon time and will be entered as covariates. Other potential confounding factors influencing platelet function (e.g. peak troponin, age, gender, diabetic status) will also be entered as covariates. Two key predictors will be tested in the model, namely the effects of (a) door-to-balloon time and (b) platelet function on arrival at the hospital.

# Study Procedures

## Patient Presentation

Patients are referred with STEMI from 3 groups; direct referral from paramedics diagnosing STEMI in the community, referral from general practice or secondary care, and referral within UHBristol (i.e. from A&E or in-patient referral).

Where possible, patients are directly transferred to the catheter laboratory and angiographic assessment is undertaken following a brief clinical assessment and consent of the patient for the procedure.

## Standard Treatment of Patients

### Pre- & Peri-Procedural Care

An oral loading dose of prasugrel is administered as soon as possible, following assessment and bivalirudin is commenced at the time of the procedure. If angiography confirms the presence of a ‘culprit’ atherosclerotic lesion within the coronary arteries, immediate PPCI is undertaken. It is usual practice for bloods to be taken from the arterial sheath at the end of the procedure to assess routine haematological and chemical pathology parameters, including: haemoglobin, white cell count and differential, platelet count, urea, creatinine, eGFR, electrolytes, liver function, CRP and troponin.

### Post-procedural Inpatient Care

Following the procedure, patients are usually transferred to the coronary care unit (CCU). Secondary preventative treatment, including beta-blockade, ACE inhibition, and statin therapy, is commenced during the in-patient stay, an echocardiographic assessment of left ventricular function is obtained and contact is made with the cardiac rehabilitation team. Ideally, patients transfer from CCU to a cardiology ward at 24hrs and discharge is arranged from 48hours onwards.

### Follow-up Care

All patients undergoing PPCI are invited to a specialised clinic at 1 month. At this appointment any ongoing symptoms/problems are assessed, a 12-lead ECG is undertaken, and a clinical examination including weight, height, heart rate, and blood pressure is performed. Where necessary, medication is up-titrated and further investigations are arranged for ongoing care.

## Research Procedures

### In-patient Procedures

Platelet function will be assessed at baseline and multiple time points after completion of the PPCI procedure, i.e. 0, 1, 2 and 24 hours. Platelet function will be assessed using a multiple electrode analyser (MEA – Multiplate platelet function analysis). This technique has been well validated for assessment of the anti-platelet effect of ADP receptor inhibitors and aspirin, offering very useful information regarding the future risk of significant adverse events(9).

MEA-derived platelet function is assessed using ‘whole blood’ samples; however, platelets are very sensitive to mechanical disruption and have a tendency to aggregate with time after obtaining the blood sample, impairing assessment. Consequently, platelet function testing requires careful sampling and timely analysis. MEA is a ‘point of care’ system and therefore we propose that the multiplate platelet function analyser will be installed in the catheter laboratory, enabling us to undertake immediate analysis of blood samples. Dr Andrew Mumford, Senior lecturer and Consultant in Haematology, has significant expertise in the field and will kindly support us in the set-up of the device.

We propose that patients will provide ‘assent’ at the time of formal consent for the PPCI procedure. The MEA testing requires: a single 3ml blood sample to be withdrawn from the arterial sheath (1x 3ml Hirudin sample), at the time of routine blood sampling, before starting the PPCI; the second sample (Time 1) will be taken at the end of the procedure; the subsequent samples (Time 2-4) will be obtained 1, 2 & 24 hours following completion of the PPCI using standard venepuncture technique. Where ever possible venepuncture will be scheduled to co-incide with clinically required phlebotomy. Immediate assessment of platelet activity (arachidonic acid, thrombin receptor, thromboxane receptor & ADP-receptor activation assessment – ASPI, TRAP, U46119 & ADP tests) will be undertaken using the ‘in-lab’ multiplate analyser. Formal written consent for measurement of platelet activity will be requested within 24 hrs of recruitment.

Clinical data (see 7.3.2 and 7.3.3) will be gathered at 24hours and at time of discharge.

### Follow-up Visits

Follow-up data will be obtained at the routine NHS follow-up provided at 1 month, as described in 8.2.3.

## Duration of Study

The full study is planned to last 24 months from 1 October 2010 to 1 October 2012. This will include a run-in period of catheter laboratory training on the multiplate analyser (5 months), recruitment (stated as 12 months), 30 day follow-up (1 month), and write up of the study (6 months).

## Data Collection

The following data will be collected at the following time-points:

| Data Item | Admission | Day 1 | Day 30 |
| --- | --- | --- | --- |
| Consent & Eligibility | + |  |  |
| CRF Data Input | + | + | + |
| Blood Sample | + (Time 0-3) | + (Time 4) |  |
| Clinical Assessment | + | + | + |

## Source Data

The following will be considered as source data:

- Participant medical notes will be the source for participant contact details, medical history and routinely collected clinical data and the clinical assessments;
- NHS laboratory databases will be the source data for blood results

The ‘in-lab’ multiplate platelet function analyser provides an averaged platelet function assessment generated from duplicate measurements. We intend to use the raw data (i.e. duplicate sample results) for analysis.

## Screening & Eligibility Assessment

Researchers will consider the eligibility criteria described in 7.4.1 & 7.4.2 before approaching the patient for consent. The patient information sheet (PIS) will be given to the patient once transferred back to CCU and a further discussion regarding the trial will be had within 24hrs. Formal written consent for participation will be sought retrospectively when the patient has had sufficient time to read and consider the PIS.

## Planned recruitment rate

The study requires serial measurement of platelet activity upto 24 hours post PPCI and therefore recruitment will be limited to weekdays between 0800-1600hrs. We anticipate completing recruitment within a 12 month period, with a minimum weekly recruitment rate of 2 patients.

## Discontinuation/Withdrawal of Participants from Study

We anticipate a follow-up rate of at least 90% for attendance at the PPCI clinic 1 month after admission. The remaining 10% of participants will be followed by telephone consultation and, if necessary, follow with their GPs to identify any important clinical events.

# Analyses

This section will be clarified and expanded in a dedicated statistics analysis plan (SAP), written before carrying out any comparative/inferential analyses and based on the standard operating procedure of the CTEU for writing and approving a SAP. The SAP will be written by a statistician during the data collection period and it will be approved by a senior statistician before databse lock and will include all analyses to be carried out.

# Project Management

The study will be managed by the Clinical Trials and Evaluation Unit (CTEU) at the Bristol Heart Institute (Co-Directors: epidemiologist/trialist, Prof B Reeves; statistician, Dr C Rogers). The CTEU will prepare all the study documentation and data collection forms, monitor recruitment, enter data and check data quality as the study progresses and carry out study analyses in collaboration with the clinical investigators.

# Safety reporting

Serious and other adverse events will be recorded and reported in accordance with the International Conference for Harmonisation of Good Clinical Practice (ICH GCP) guidelines and the Sponsor’s Research Related Adverse Event Reporting Policy. However, the only research procedures that patients are required to undergo for this study are venipuncture (for blood samples).

We do not expect any adverse events as a result of this study.

# Ethical considerations

## Ethical review

Ethics review of the protocol for the study and other study-related essential documents (e.g. PIS, consent form) will be carried out by a UK NHS Research Ethics Committee (REC) and other bodies with similar roles/authority for centres outside the UK. This study does not raise any substantive ethical issues since participants will be receiving standard NHS care and having research investigations that carry very little risk.

## Obtaining informed consent from participants

Due to the time-critical nature of treatment for STEMI it is not possible to obtain informed consent prior to recruitment into the trial and collection of blood samples during the index PPCI procedure. Therefore, we intend to obtain ‘assent’ for enrolment into the study at the time of obtaining procedural consent in the catheterisation laboratory.

A member of the research team will further inform the participant about the study following transfer to the CCU. Written information defining the study design, protocol and demands on participants will be given to patients, in the form of a patient information sheet (PIS). Informed consent will be obtained following a minimum of 4hours to consider the information offered and having been invited to ask further questions about the study.

# Research governance

The University Hospitals Bristol NHS Foundation Trust (UHBristol) is the sponsor for the study.

This study will be conducted in accordance with:

- The European Union Directive 2001/20/EC on clinical trials;
- The Medicine for Human Use (Clinical Trial) Regulations 2004;
- International Conference for Harmonisation of Good Clinical Practice (ICH GCP) guidelines;
- Research Governance Framework for Health and Social Care.

## NHS approval

The investigators will seek approval to carry out the study from the University Hospitals of Bristol NHS Trust. Local Research and Development (R&D) approval in the UK requires that the study be conducted in compliance with the Research Governance Framework.

## Investigators' responsibilities

Investigators will be required to ensure that NHS Research Ethics Committee (REC) and research governance approvals have been obtained and that any contractual agreements required have been signed off by all parties prior to the start of the study. Investigators will be required to ensure compliance to the protocol and study manual and with completion of the CRFs. Investigators will be required to allow access to study documentation or source data on request for monitoring visits and audits performed by the Sponsor or CTEU or any regulatory authorities.

## Monitoring by sponsor

The study will be monitored and audited in accordance with the Sponsor’s policy, which is consistent with the Research Governance Framework and the Medicines for Human Use (Clinical Trials) Regulations 2004. All study related documents will be made available on request for monitoring and audit by the sponsor (or CTEU if they have been delegated to monitor) or the Ethics Committee.

## Indemnity

This is an NHS-sponsored research study. For NHS sponsored research, HSG(96)48 reference no.2 refers. If there is negligent harm during the clinical study when the NHS body owes a duty of care to the person harmed, NHS Indemnity covers NHS staff, medical academic staff with honorary contracts, and those conducting the study. NHS Indemnity does not offer no-fault compensation and is unable to agree in advance to pay compensation for non-negligent harm. Ex gratia payments may be considered in the case of a claim

# Data protection and patient confidentiality

Data will be collected and retained in accordance with the UK Data Protection Act 1998.

## Data handling

Data will be entered onto a database and data validation and cleaning will be carried out throughout the study.

## Data storage

We will propose to the NHS REC that we retain all study documentation in a secure location during the conduct of the study and for 5 years after the end of the study, when all patient identifiable paper records will be destroyed by confidential means. In compliance with the MRC Policy on Data Preservation, we will also propose that the fully anonymised dataset, a separate secure electronic ‘key’ with a unique patient identifier, and relevant ‘meta’-data about the study be retained in electronic form indefinitely because of the potential for the raw data to be used subsequently for secondary research.

## Data sharing

Data will not be made available for sharing until after publication of the main results of the study. Thereafter, anonymised individual patient data will be made available for secondary research, conditional on assurance from the secondary researcher that the proposed use of the data is compliant with the MRC Policy on Data Preservation and Sharing regarding scientific quality, ethical requirements and value for money. We propose that a minimum requirement with respect to scientific quality should be a publicly available pre-specified protocol describing the purpose, methods and analysis of the secondary research, e.g. a protocol for a Cochrane systematic review. The second file containing patient identifiers would be made available for record linkage or a similar purpose, subject to confirmation that the secondary research protocol has been approved by a UK REC or other similar, approved ethics review body.

# Dissemination of findings

The findings will be disseminated by usual academic channels, i.e. presentation at international meetings, as well as by peer-reviewed publications and through patient organisations and newsletters to patients, where available.

# References
